# How Carborundum Is Made

**Published:** 1896-06

**Authors:** 


### Selections.

### How Carborundum is Made.

It could not have been made at all—this compound of carbon and silicon which is coming into such universal use as an abrasive —were it not for the electric furnace, with its degree of heat that was quite undreamed of only a few years ago. Discovered by accident in experiments on electric smelting, it is now made in large quantities, and its manufacture is one of the first to employ the electric energy generated by the world-famous power-plant at Niagara. We quote a few paragraphs, telling how this power is utilized, from *The Electrical World* October 26th. The crude materials, we are told at the outset, are simple enough, being nothing but coke, sand, salt, and sawdust, which are first thoroughly ground and mixed. We now let the article speak for itself:

"The four crude materials having been thus thoroughly mixed, the product is conveyed to the electrical furnaces, situated in an adjoining building, and which have the appearance of rough and apparently crude, oblong brick boxes, made without cement, mortar or other binding materials. Provision is made for five of these boxes, which extend down one side of the large spacious building, each of them measuring about 15 feet in length by 7 feet in width and the same in height. In the center of each end is placed a large bronze plate, and these are connected by means of four large copper cables to massive copper bars extending under the floor at either end of the furnaces. Connecting with the inner surfaces of the bronze plates are 120 carbon rods, 60 to each plate. These carbon rods are three inches in diameter and something over two feet in length, and are so placed as to pass through the end walls of the brick box or furnace, projecting into the interior and toward each other, thus constituting the terminals. Into this rectangular brick box the mixture that has been prepared in the stock room is introduced, about ten tons constituting a charge, and through the center of the mass of mixed materials is placed a core or cylinder of granules of crushed coke extending from the carbon rods at one end of the furnace to those at the other end, a perfect electrical connection through the furnace, by means of the bronze plates, carbon rods, and the core, being thus made."

Into this furnace is turned a powerful electric current of 1,C00 horse-power, all of which is transformed within it into heat. The results are thus described:

"A short time, perhaps two hours, after the turning on of the current, gases begin to escape through the crevices of the brick walls of the furnace, and, being ignited, burn with a lambent blue flame. As the process continues the outer walls and top of the mass in the furnace slowly rises in temperature through the transmission of the intense heat from the core, the entire top of the mass being red-hot in about 12 hours. After the current has remained on for the period of 24 hours, or until such time as the workman in charge recognizes as sufficient, it is switched off in the transformer building, the flexible cables are disconnected from the bronze plates and others are connected with the plates of the next furnace in the series of five, which in turn are carried through the same operation.

"One end of the first furnace is then removed and a cross-section through its center exposed, thus permitting of a ready inspection of the results of the operation. In the center is the granular core, in the same position in which it was originally placed, but it is now purified of all foreign substances. It is now pure carbon and has lost about one-fourth of its weight; this loss represents the volatilized impurities. The presence of grains of graphite disseminated throughout its mass indicate that its temperature must have been near 7,000 degrees F.—the point of graphic formation. Surrounding the core in the form of a cylinder is a beautiful crystalline formation, the crystals being constructed on lines radiating from the center. The crystals in immediate contact with the core are looped or built together into one concrete mass; as the distance from the core is increased, the size of the crystals diminishes rapidly, until at about 15 inches all crystallization cases and an amorphous material is encountered, of a whitish-gray color, for a distance of two inches, when a sudden change occurs to a black mass composed of the original mixture, now held together in a cemented state by the fusion of the salt. The crystalline and amorphous material, lying between the core and the outer black mass is carbid of silicon, being composed of equal atoms of carbon and silicon. About two tons of carborundum is produced in one furnace run, and to prepare it for the market it is first passed under heavy iron rolls for the purpose of crushing apart and separating the individual crystals, after which it is treated with an acid and water-bath to remove solubles. It is then dried and sifted, to separate the various sizes."

Of the uses of the compound thus made—which, it may be said in passing, is so hard that it will scratch any other substance except the diamond—we are told, at the close of the article:

"Owing to the limited facilities heretofore existing, the production of carborundum has been so small—not over 300 pounds per day—as to practically restrict its uses to the finer trades, such as the dental and manufacturing jewelers' trades, fine-tool grinding, pearl-grinding and kindred industries. The development in the dental trade especially has been remarkable, and in the form of disks, lathe and engine wheels and cloth-finishing strips, carborundum is rapidly displacing all other abrasive substances in this important industry, not only in the United States but throughout Europe."—*The Literary Digest.*

